# Gene Co-expression Analysis of the Human Substantia Nigra Identifies ZNHIT1 as an *SNCA* Co-expressed Gene that Protects Against α-Synuclein-Induced Impairments in Neurite Growth and Mitochondrial Dysfunction in SH-SY5Y Cells

**DOI:** 10.1007/s12035-022-02768-9

**Published:** 2022-02-17

**Authors:** Erin McCarthy, Aaron Barron, Noelia Morales-Prieto, Martina Mazzocchi, Cathal M. McCarthy, Louise M. Collins, Aideen M. Sullivan, Gerard W. O’Keeffe

**Affiliations:** 1 Department of Anatomy & Neuroscience, University College Cork, Cork, Ireland; 2 Department of Pharmacology and Therapeutics, University College Cork, Cork, Ireland; 3 Department of Physiology, University College Cork, Cork, Ireland; 4 Parkinson’s Disease Research Cluster (PDRC), University College Cork, Cork, Ireland; 5 APC Microbiome Ireland, University College Cork, Cork, Ireland

**Keywords:** Parkinson’s disease, α-Synuclein, SNCA, ZNHIT1, Dopamine, Dopaminergic, Substantia nigra, Histone deacetylase, Axon degeneration, Histone acetylation

## Abstract

Parkinson’s disease (PD) is neurodegenerative disorder with the pathological hallmarks of progressive degeneration of midbrain dopaminergic neurons from the substantia nigra (SN), and accumulation and spread of inclusions of aggregated α-synuclein (α-Syn). Since current PD therapies do not prevent neurodegeneration, there is a need to identify therapeutic targets that can prevent α-Syn-induced reductions in neuronal survival and neurite growth. We hypothesised that genes that are normally co-expressed with the α-Syn gene (*SNCA*), and whose co-expression pattern is lost in PD, may be important for protecting against α-Syn-induced dopaminergic degeneration, since broken correlations can be used as an index of functional misregulation. Gene co-expression analysis of the human SN showed that nuclear zinc finger HIT-type containing 1 (ZNHIT1) is co-expressed with *SNCA* and that this co-expression pattern is lost in PD. Overexpression of ZNHIT1 was found to increase deposition of the H2A.Z histone variant in SH-SY5Y cells, to promote neurite growth and to prevent α-Syn-induced reductions in neurite growth and cell viability. Analysis of ZNHIT1 co-expressed genes showed significant enrichment in genes associated with mitochondrial function. In agreement, bioenergetic state analysis of mitochondrial function revealed that ZNHIT1 increased cellular ATP synthesis. Furthermore, α-Syn-induced impairments in basal respiration, maximal respiration and spare respiratory capacity were not seen in ZNHIT1-overexpressing cells. These data show that ZNHIT1 can protect against α-Syn-induced degeneration and mitochondrial dysfunction, which rationalises further investigation of ZNHIT1 as a therapeutic target for PD.

**Supplementary Information:**

The online version contains supplementary material available at 10.1007/s12035-022-02768-9.

## Introduction

Parkinson’s disease (PD) is a neurodegenerative disorder that is characterised by progressive loss of midbrain dopaminergic neurons from the *substantia nigra* (SN) [1, 2]. The axons of these neurons form the nigrostriatal pathway, which innervates the striatum and is a key part of the basal ganglia circuitry which regulates voluntary movement [1, 2]. Dysfunction of this nigrostriatal pathway resulting from its progressive degeneration in PD leads to motor impairments including akinesia, bradykinesia and resting tremor, which are the core clinical features of this disease [3, 4].

PD is a synucleinopathy characterised by the accumulation of intracellular proteinaceous aggregates, called Lewy bodies and Lewy neurites, which consist predominantly of the protein α-synuclein (α-Syn), which is encoded by the *SNCA* gene [5–7]. A definitive link between *SNCA* and PD came with the demonstration that mutations [8, 9], or duplications and triplications [10, 11], in *SNCA* cause autosomal-dominant PD and that genetic polymorphisms in *SNCA* are risk factors for sporadic PD [12–16]. Moreover, rodent neurons overexpressing αSyn [17], or iPSC-derived dopaminergic neurons carrying mutations, and duplications or triplications of *SNCA* have reduced survival and defects in neurite growth [18, 19]. In agreement, several studies have shown nigrostriatal degeneration in rodents and non-human primates, resulting from injection of α-Syn pre-formed fibrils (PFFs) or recombinant adeno-associated virus vector (rAAV)-mediated α-Syn overexpression (for review see [20]). Collectively, these data show that α-Syn accumulation plays a role in dopaminergic degeneration in PD. Therefore, it is important to investigate the genes and molecular networks that are affected by α-Syn, in order to elucidate the molecular basis of cellular dysfunction in PD and to identify new therapeutic targets.

Gene co-expression analysis is an approach that can be used to associate genes of unknown function with specific biological processes, to prioritise candidate disease genes or to discern transcriptional regulatory programmes [21, 22]. In this study, we performed gene co-expression analysis in which we examined all possible pairwise correlations between *SNCA* and other genes in SN, hippocampus and occipital cortex of a human brain transcriptome dataset [23], to identify genes that are uniquely co-expressed with *SNCA* in the SN. Following an enrichment analyses, we selected genes for functional analysis depending upon whether their co-expression with *SNCA* was altered in PD; since normal co-expression patterns tend to break down in disease states, these broken correlations can be used as an index of functional misregulation [24–26].

We found that the gene z*inc finger HIT-type containing 1* (*ZNHIT1*) is co-expressed with the *SNCA* gene and that this co-expression in the SN is lost in PD. ZNHIT1 is a core component of the SNF2-related CBP activator protein (SRCAP) complex, which is an ATP-dependent chromatin remodelling complex that replaces histone (H)2A with the histone variant H2A.Z in the nucleosome to regulate gene expression [27, 28]. Previous studies have shown that ZNHIT1 regulates gene expression associated with mitochondrial function during prenatal cardiac development [29] and controls intestinal stem cell maintenance by regulating H2A.Z incorporation [30]. However its function in the neural cells and in the nervous system is largely unknown.

## Materials and Methods

### Gene Expression Analysis of Human SN

Gene expression data for healthy control human SN (GSE:60,863 [31]) were analysed using R2 Genomics Analysis and Visualization Platform [32]. This gene expression data from the GSE:60,863 dataset was generated by the UK Brain Expression Consortium. It contains gene expression data from *post mortem* human brain samples that were dissected from ten different brain regions. A total of 1231 samples were obtained from 134 Caucasian neuropathologically and neurologically normal individuals; the aim of that study was further understanding of gene expression regulation in the human brain [31]. Gene set enrichment analysis was carried out using STRING (https://string-db.org) and PANTHER (http://pantherdb.org) to assess which genes were significantly co-expressed with *SNCA* in the SN. All gene expression data are presented as log2 expression values. Gene ontology (GO) enrichment analysis was performed using the gene ontology platform (http://geneontology.org/).

### Cell Culture

Human SH-SY5Y cells (ATCC; CRL-2266) are extensively used as models of human DA neurons [33]. SH-SY5Y cells were generally plated at a density of 5 × 10^5^ cells per well in a 24-well plate and grown in Dulbecco’s modified eagle medium nutrient mixture F-12 (Sigma) with 10% foetal calf serum (Sigma), supplemented with 100 nM L-glutamine (Sigma), 100 U/ml penicillin (Sigma) and 10 µg/ml streptomycin (Sigma). These cells were incubated in a humified atmosphere containing 5% CO_2_ at 37 °C. A differentiating agent was not applied as the SH-SY5Y cells develop clear neurites when cultured at low density. Furthermore, differentiating agents may cause them to become less susceptible to the effects of *SNCA*. SH-SY5Y cells were fixed in 4% paraformaldehyde at 72 h post-transfection.

### Plasmid Transfection

SH-SY5Y cells were transfected using the TransIT-X2® Dynamic Delivery System (Mirus Bio, Cat # 6000) as per the manufacturer’s instructions. SH-SY5Y cells were seeded at a density of 5 × 10^5^ cells per well in a 24-well plate. Where indicated, cells were transfected at 1 DIV with varying combinations of plasmids, as indicated in each figure legend: pcDNA3-EGFP (Addgene plasmid # 13,031; a gift from Douglas Golenbock), EGFP-alpha-synuclein-WT (Addgene plasmid # 40,822; a gift from David Rubinsztein [34]), FLAG-ZnF/HIT1 (Addgene plasmid # 15,332, a gift from Joan Conaway and Ronald Conaway [35]), pT-FLAG (Addgene plasmid # 31,385; a gift from Yegor Vassetzky [36]) and GFP Cignal reporter (Qiagen CCS-017G). Five hundred nanograms of each plasmid was mixed with 1.5 µl of TransITX2® in 50 µl of media and incubated for 30 min at room temperature, before being added to the cultures.

### Immunocytochemistry and Analysis of Neurite Growth

Prior to imaging, cells were fixed for 25 min in 4% paraformaldehyde, followed by 3 × 5-min washes in 0.02% Triton X-100 in 10 mM phosphate-buffered saline (PBS-T). The cells were then incubated in 5% bovine serum albumin (BSA) in 10 mM PBS-T for 1 h at room temperature. They were then incubated in one of the following antibodies: phospho-Smad 1/5/9 (Cell Signaling 13820S; 1:500), AcH3 K9-K14 (Santa Cruz sc-33361; 1:500) or ZNHIT1 (Thermo Fisher # PA5-53,903; 1:500), diluted in 1% BSA in 10 mM PBS at 4 °C for 16 h. Following 3 × 5-min washes in 10 mM PBS-T, cells were incubated in 594-conjugated Alexa Fluor® secondary antibody (Invitrogen; 1:500 A11005 or A11012) in 1% BSA in 10 mM PBS, prior to 3 × 5-min washes. For analysis of neurite growth, six non-overlapping images were captured from each well in each experimental group, using an Olympus IX71 inverted microscope. Neurite growth was measured by opening each image in Image J and manually tracing the length of a given neurite using the trace function in Image J. Where indicated, the fluorescence intensity of individual cells was measured by densitometry using Image J analysis software. Specifically, fluorescence intensity was measured using the corrected total cell fluorescence (CTCF) method, which determines the corrected total fluorescence by subtracting the background signal and taking into account the area of the fluorescing cell. The formula used to calculate the fluorescence intensity of individual cells was (CTCF) = Integrate Density – (Area of Selected Cell × Mean Fluorescence of Background). These individual cell values were then averaged to get one value per group per N.

### Generation of Stable GFP and α-Syn-GFP SH-SY5Y Cell Lines

For generation of stable cell lines, SH-SY5Y cells were plated at the density of 4 × 10^5^ cells in a T-75 culture flask and transfected using TransIT-X2® Dynamic Delivery System (Mirus Bio, Cat # 6000) as per the manufacturer’s instructions, with 15 μg of pcDNA3-EGFP (Addgene plasmid # 13,031; a gift from Professor Douglas Golenbock) or of EGFP-alphasynuclein-wild-type (Addgene plasmid # 40,822). At 72 h post-transfection, cells were exposed to 200 mg/ml G418 (Sigma) allowing selection based on plasmid-specific antibiotic resistance. Stably-transfected cells were split weekly for 4 weeks, with addition of G418 until visual confirmation of fluorescent EGFP expression in all cells.

### Seahorse Assay to Assess Mitochondrial Function

Mitochondrial function and metabolism were assessed using the Seahorse XF96 Mito Stress Test (Agilent Technologies). The stable GFP or α-Syn-GFP SH-SY5Y cell lines were seeded at 4 × 10^5^ cells/well in a XF96 culture plate and transfected 24 h later with 500 ng of plasmid carrying either FLAG (Addgene #31,385) or FLAG-tagged ZNHIT1 (Addgene #15,332) for 72 h. At 1 h before the assay, the media was changed to Seahorse XF DMEM media, supplemented with 2 mM L-glutamine, 1 mM pyruvate and 10 mM glucose, and cells were allowed to equilibrate at 37 °C and 0% CO_2_ for 1 h. After calibration, oxygen consumption rate (OCR) was measured by the Seahorse XF96 Analyzer and recorded with XF Wave software 1.4.2. at 12 timepoints over the 80-min run: three times at basal respiration, three times after injection of 2.5 μM oligomycin to inhibit complex V, three times after injection of 2 μM of the ionophore carbonyl cyanide-p-trifluoromethoxyphenylhydrazone (FCCP) to depolarize the inner mitochondrial membrane and three times after injection of 0.5 μM each of rotenone and antimycin A, to inhibit complexes I and III, respectively. After completion of the assay, cells were lysed in 1 × RIPA buffer, and total protein was quantified by bicinchoninic acid (BCA) assay. OCR values normalised to the amount of protein per well. From normalised OCR values, the following respiratory parameters were calculated: basal respiration, proton leak, maximal respiration, non-mitochondrial respiration, ATP production and spare respiratory capacity.

### Statistical Analysis

Statistical analysis was performed using GraphPad Prism 9 (©2021 GraphPad Software, CA USA). All data are presented as the mean ± SEM of the number of experimental replicates rather than the number of cells. Statistical differences were analysed using two-way ANOVA as appropriate, with post hoc tests as indicated in the figure legends.

## Results

### Identification of SNCA Co-expressed Genes in the Human SN Reveals Enrichment of Genes Involved in Histone Deacetylation, and Altered ZNHIT1-SNCA Co-expression in the SN in PD

**Fig. 1:**
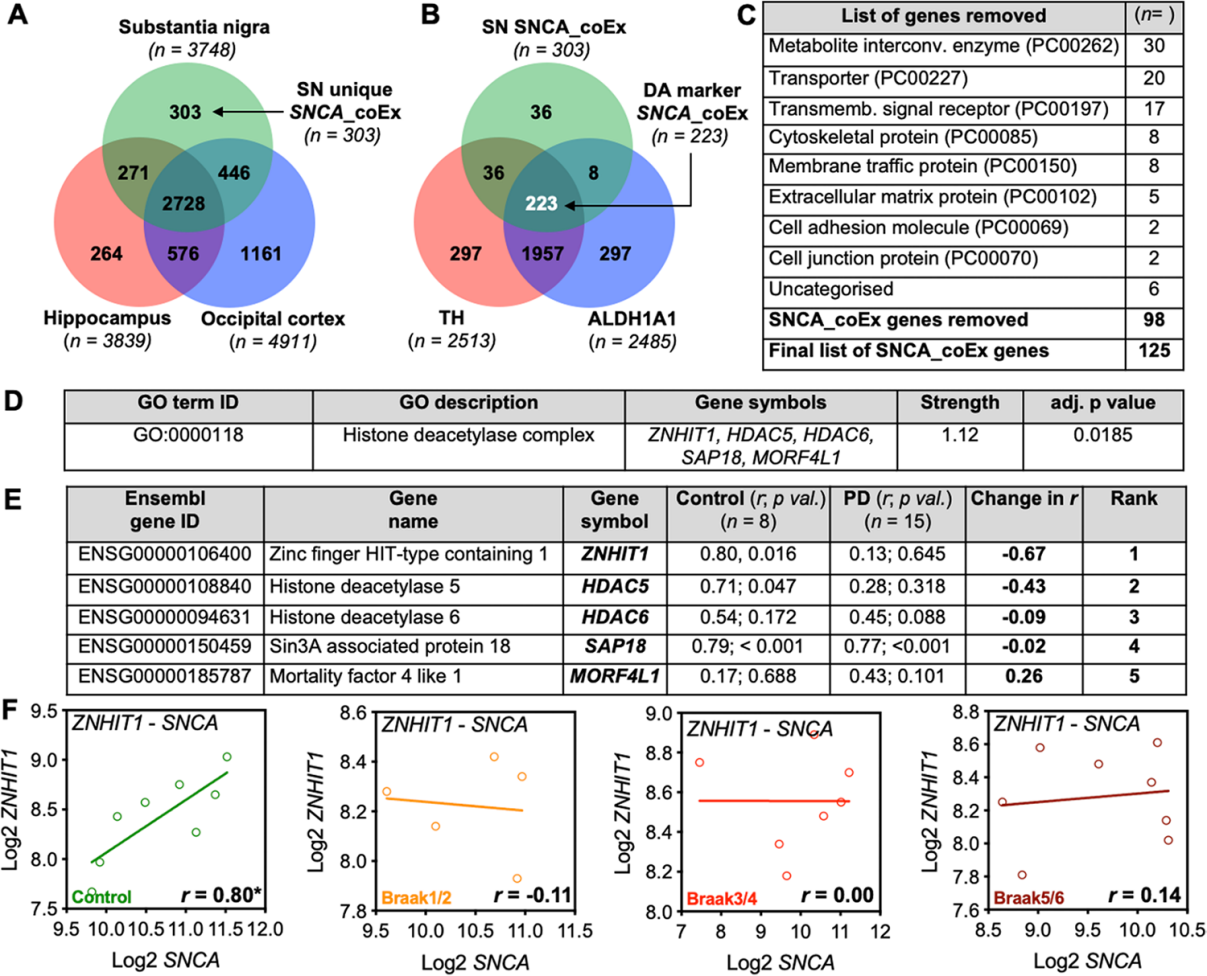
Identification of *SNCA* co-expressed genes in the human SN reveals enrichment of genes involved in histone deacetylation, and altered *ZNHIT1-SNCA* co-expression in the SN in PD.** A** Venn diagram showing the overlap between genes with positive significant co-expression with *SNCA* in the SN (green), hippocampus (red) and occipital cortex (blue), after Bonferroni correction. This analysis identified *n* = 303 *SNCA* co-expressed (SNCA_coEx) genes that were unique to the SN. **B** Venn diagram showing the overlap between SNCA_coEx genes (green) and genes co-expressed with the dopaminergic markers *TH* (red) and *ALDH1A1* (blue) in the SN. This analysis identified *n* = 223 *SNCA* co-expressed (SNCA_coEx) genes that overlapped with *TH* and *ALDH1A1* co-expressed genes. In **A** and **B** the total number of genes in each case is shown in parentheses. Raw data was derived from GSE60863. **C** The list of *n* = 223 genes were classified using the Panther classification tool (http://pantherdb.org), and the table shows the list of functional and/or structural gene classes that were removed from the *n* = 223 genes to generate a final list of *n* = 125 SNCA_coEx genes in the SN. **D** Results of the gene ontology (GO) enrichment analysis of the *n* = 125 genes, which was performed using STRING (https://string-db.org). The *p* value was corrected for multiple testing using the Benjamini–Hochberg procedure.** E** Table showing the changes in the co-expression pattern (*r2* – *r*1) of *SNCA* with *ZNHIT1*, *HDAC5*, *HDAC6*, *SAP18* and *MORF4L1* in control (*n* = 8) and PD (*n* = 15) SN samples. **F** Linear regression analysis showing the correlations between *ZNHIT1* and *SNCA* in control (*n* = 8), Braak stage 1/2 (*n* = 5), Braak stage 3/4 (*n* = 7) and Braak stage 5/6 (*n* = 8) SN samples. The *r* values are shown on each linear regression graph. Raw data was derived from GSE49036. All analyses were performed using the R2 genomics analysis and visualisation platform (https://hgserver1.amc.nl/cgi-bin/r2/main.cgi)

We first sought to identify *SNCA* co-expressed genes that were enriched in the SN, by performing pair-wise correlations between *SNCA* and all other genes expressed in the human SN (*n* = 101 samples), hippocampus, (*n* = 122) and occipital cortex (*n* = 122) using open-source human brain transcriptome data (GSE:60,863) [23]). This analysis identified *n* = 303 genes that were co-expressed with *SNCA* and that were unique to the SN (Fig. 1A). We next enriched this gene list for those most likely to be expressed in dopaminergic neurons by comparing the *n* = 303 list to those genes that were co-expressed with two dopaminergic markers, *TH* and *ALDH1A1*, in the SN. We found that *n* = 233 *SNCA* co-expressed genes that were enriched in the SN were also co-expressed with *TH* and *ALDH1A1* (Fig. 1B). We next used PANTHER (http://pantherdb.org) to perform a protein classification analysis, to group genes into categories in order to remove those genes involved in general aspects of cellular function (Fig. 1C); this generated a final list of *n* = 125 genes. We next performed a gene set enrichment analysis using STRING (https://string-db.org) and found a significant enrichment of genes associated with the histone deacetylase complex (GO:0,000,118); these were *ZNHIT1*, *HDAC5*, *HDAC6*, *SAP18* and *MORF4L1* (Fig. 1D).

We next used available transcriptome data from the SN of age- and gender-matched control and PD samples (GSE49036) [37] to examine the co-expression of the five lead genes identified from our earlier co-expression analysis with *SNCA* in the SN in healthy controls and subsequently to determine whether their co-expression pattern with *SNCA* is altered in PD. The rationale for doing this is that genes with a functional association are strongly co-expressed, and these normal co-expression patterns tend to break down in a disease state; therefore, broken correlations can be used as an index of functional misregulation [24–26]. To do this, we used open source transcriptome data (GSE 49,036) from a previously published report [37] that had performed microarray analysis on SN samples taken from controls (*n* = 8) and patients with PD (*n* = 15). Of the 5 lead genes identified from our earlier analysis (*ZNHIT1*, *HDAC5*, *HDAC6*, *SAP18*, *MORF4L1*), we validated a significant correlation between *ZNHIT1*, *SAP18* and *HDAC5* with *SNCA* in the SN of controls (Fig. 1E). In contrast, while the significant positive correlation between *SAP18* and *SNCA* was maintained in PD (*r* = 0.77, *p* = 6.51 × 10^−04^), the correlations of both *ZNHIT1* (*r* = 0.13, not significant (n.s.)) and *HDAC5* (*r* = 0.28, n.s.) with *SNCA* was lost in PD SN samples, with the greatest change in *r* seen in *SNCA-ZNHIT1* (Fig. 1E). Since HDAC5 has previously been shown to regulate α-Syn-induced impairments in neurite growth [38, 39], we focused on ZNHIT1 in subsequent analysis, as the aim was to identify novel genes that may be relevant to α-Syn-induced impairments in cellular function.

We next investigated the correlation between the top ranked gene, *ZNHIT1*, and *SNCA* in the SN of samples classified as controls (*n* = 8), Braak stage 1/2 (*n* = 5), Braak stage 3/4 (*n* = 7) and Braak stage 5/6 (*n* = 8) PD, to investigate at which disease state the co-expression patterns break down. We found a significant correlation between *ZNHIT1* and *SNCA* (*r* = 0.80, *p* = 0.0160) in control samples; however, this correlation was lost even at the earliest Braak stages 1/2 (*r* =  − 0.11, n.s.) and at all stages thereafter (Fig. 1F). These broken correlations suggest that there is an early and sustained functional dysregulation of *SNCA-ZNHIT1* in PD.

### ZNHIT1 Increases Incorporation of the Histone Variant H2A.Z in SH-SY5Y Cells and This Is Unaffected by α-Synuclein Overexpression

ZNHIT1 has been shown to regulate H2A.Z deposition and thereby modulate gene transcription [40]. H2A.Z incorporation regulates important cellular and molecular processes including heterochromatin regulation [41], DNA repair [42, 43] and transcriptional regulation [44]. Additionally, H2A.Z is incorporated into the SRCAP complex [45], an ATP-dependent complex involved in the regulation of chromatin remodelling, of which ZNHIT1 is the main regulatory component [46].

**Fig. 2:**
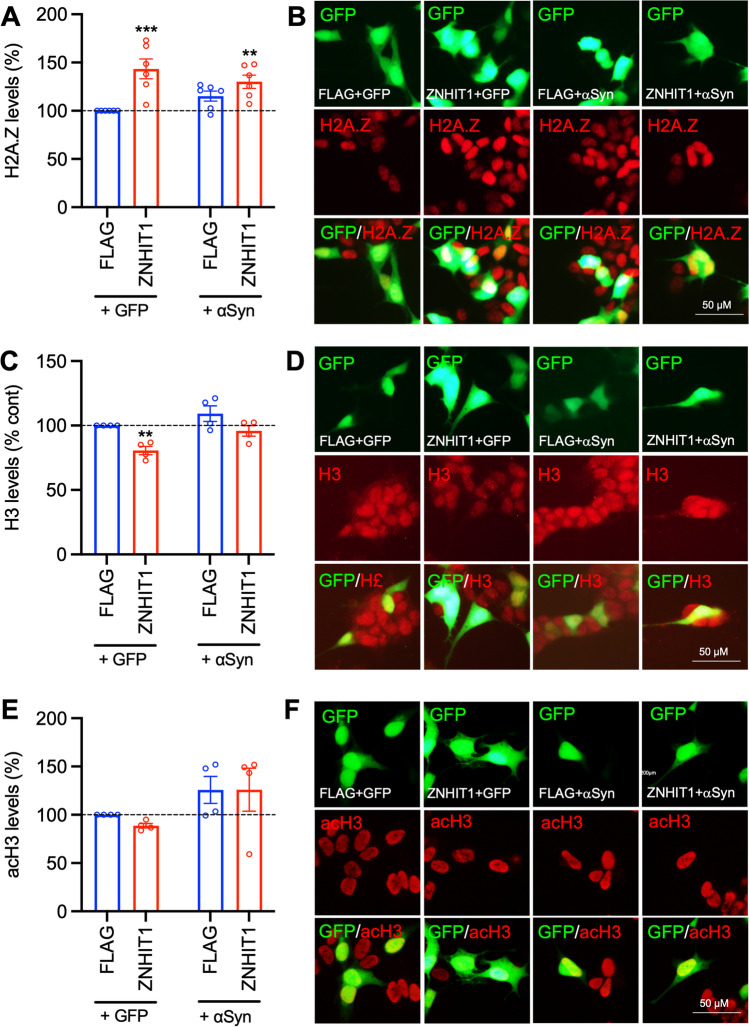
ZNHIT1 increases incorporation of the histone variant H2A.Z in SH-SY5Y cells and this is unaffected by *α*-synuclein overexpression. **A**–**F** SH-SY5Y cells were transfected with 500 ng of plasmid expressing either FLAG (Addgene #31,385) or FLAG-tagged ZNHIT1 (Addgene #15,332), together with a plasmid expressing either GFP or GFP-tagged wild-type α-synuclein (αSyn) (Addgene #40,824). All analyses were performed at 72 h post-transfection. **A**–**F** Graphs and representative photomicrographs of **A**, **B** H2A.Z, **C**, **D** H3 and **E**, **F** acetylated (ac)H3 levels in transfected (GFP +) cells. All data are mean ± SEM expressed as a percentage of the control (FLAG + GFP) from *n* = 3–6 independent experiments. Two-way ANOVA with post hoc Fishers LSD test (**p < 0.01, ***p < 0.001 vs. FLAG + GFP)

Based on this, we investigated the effects of ZNHIT1 and α-Syn overexpression on incorporation of H2A.Z in SH-SY5Y cells. SH-SY5Y cells were transfected with plasmids expressing either GFP or GFP-tagged wild-type human α-Syn, together with either FLAG (control) or FLAG-tagged human ZNHIT1 and found them to be co-transfected (Supplementary Fig. 1). The levels of H2A.Z, H3 and acH3 protein expression in GFP + -transfected cells were measured at 72 h post-transfection using immunocytochemistry and densitometry. Two-way ANOVA revealed a significant effect of ZNHIT1 (*F*_1,20_ = 18.97, *p* = 0.0003) on H2A.Z expression in SH-SY5Y cells (Fig. 2A, B). Post hoc testing revealed a significant increase in H2A.Z expression in the ZNHIT1 + GFP group compared to the control group (Fig. 2A, B). Furthermore, there was a significant increase in H2A.Z expression in the ZNHIT1 + α-Syn group compared to the control group, and no significant increase over control was seen in the FLAG + α-Syn group (Fig. 2A, B). These data show that ZNHIT1 increases H2A.Z incorporation, even when co-expressed with α-Syn.

We next examined the levels of H3 and levels of acetylated H3 (acH3), as *α*-Syn has been shown to act within the nucleus to inhibit the acetylation of histone 3 (H3) and thus to promote neurotoxicity in SH-SY5Y cells and in wild-type *α*-Syn transgenic *Drosophila* [47]. SH-SY5Y cells were transfected with plasmids expressing either GFP or GFP-tagged wild-type human α-Syn, together with either FLAG or FLAG-tagged human ZNHIT1, and immunocytochemistry was performed for H3 or acH3 at 72 h post-transfection. Two-way ANOVA revealed a significant main effect of ZNHIT1 (*F*_1, 12_ = 16.86, *p* = 0.0015) on H3 levels (Fig. 2C, D). Post hoc testing showed that overexpression of ZNHIT1 significantly reduced cellular levels of H3, whereas no significant reduction from control levels was seen in the FLAG + α-Syn group (Fig. 2C, D). Cellular levels of acH3 were unaffected by the overexpression of ZNHIT1 (*F*_1,12_ = 0.177, n.s.) (Fig. 2D, E). Collectively, these data suggest that ZNHIT1 overexpression regulates cellular levels of H2A.Z and H3, while ZNHIT1-induced reductions in H3 do not occur in the presence of αSyn.

### ZNHIT1 Promotes Neurite Growth and Protects Against α-Synuclein-Induced Reductions in Neurite Growth and Cell Viability in SH-SY5Y Cells

**Fig. 3:**
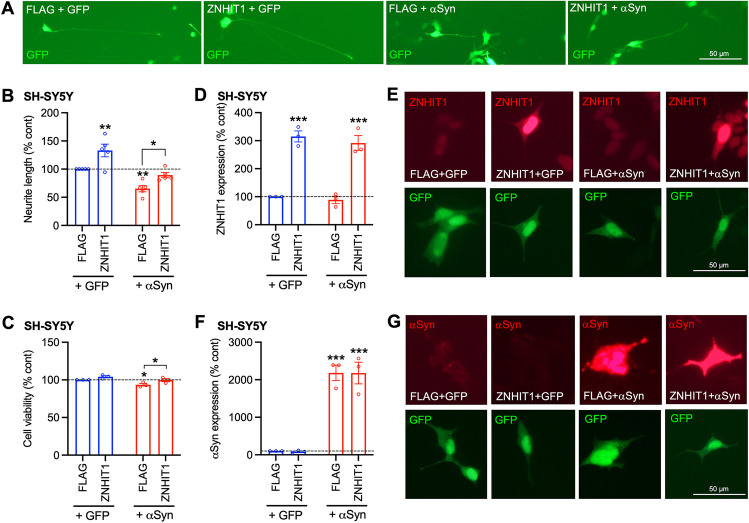
ZNHIT1 promotes neurite growth and protects against *α*-synuclein-induced reductions in neurite growth and cell viability in SH-SY5Y cells. **A** Representative photomicrographs, **B** graph of neurite length and **C** graph of cell viability of SH-SY5Y cells transfected with 500 ng of a plasmid expressing either FLAG (Addgene #31,385) or FLAG-tagged ZNHIT1 (Addgene #15,332), together with a plasmid expressing either GFP or GFP-tagged wild-type α-synuclein (αSyn) (Addgene #40,824). All analyses were performed at 72 h post-transfection. **D**, **E** Graph and representative photomicrographs showing the relative levels of ZNHIT1 (red) in transfected GFP + cells (green). **F**, **G** Graph and representative photomicrographs showing the relative levels of αSyn (red) in transfected GFP + cells (green). All data are mean ± SEM expressed as a percentage of the control (FLAG + GFP) from *n* = 3–6 independent experiments. Two-way ANOVA with post hoc Fishers LSD test (* *p* < 0.05, ** *p* < 0.01, *** *p* < 0.001 vs. FLAG + GFP or as indicated)

We next sought to investigate the potential functional role of ZNHIT1 in a cellular model of *α*-synucleinopathy; we used human SH-SY5Y cells, which are widely used to study molecular and cellular mechanisms of relevance to PD [48]. SH-SY5Y cells were transfected with plasmids expressing either GFP or GFP-tagged wild-type human *α*-Syn, together with either FLAG or FLAG-tagged human ZNHIT1. We examined neurite growth in individual GFP + transfected SH-SY5Y cells at 72 h post-transfection, to determine whether ZNHIT1 expression could alter the known inhibitory effect of *α*-Syn on neurite growth in these cells [39]. Two-way ANOVA revealed a significant effect of *α*-Syn (*F*_1,16_ = 34.43, *p* = 0.0001) and of ZNHIT1 (*F*_1,16_ = 18.34, *p* = 0.0006) on neurite growth (Fig. 3A, B). Post hoc testing revealed a significant increase in basal levels of neurite growth in the ZNHIT1 + GFP group compared to the control group (Fig. 3A, B). Furthermore, there was a significant reduction in neurite growth in the FLAG + *α*-Syn group that was not seen in the ZNHIT1+*α*-Syn group (Fig. 3A, B).

Having demonstrated the beneficial effects of ZNHIT1 on neurite growth, we next examined the effects of ZNHIT1 and *α*Syn on cell viability, which was measured using MTT assay at 72 h post-transfection. Two-way ANOVA revealed a significant effect of *α*-Syn (*F*_1,8_ = 10.97, *p* = 0.0107) and ZNHIT1 (*F*_1,8_ = 12.48, *p* = 0.0077) on cell viability (Fig. 3C). Post hoc testing revealed a significant reduction in neurite growth the FLAG + *α*-Syn group compared to the control that was not seen in the ZNHIT1+*α*-Syn group (Fig. 3C). Although the magnitude of these changes were small in the current experiment, it is important to note that the transfection efficiency was approximately 30% which is likely to mask the true extent of any effect in these cell viability experiments.

To ensure that these effects on neurite growth and cell viability were not due to different levels of ZNHIT1 or *α*-Syn between the groups, we quantified the expression of these proteins by immunocytochemistry followed by densitometry in transfected (GFP +) cells. These analyses showed equal levels of expression of ZNHIT1 in both groups transfected with ZNHIT1 (Fig. 3D, E), and equal levels of expression of *α*-Syn in both groups transfected with *α*-Syn (Fig. 3F, G). Moreover, in the basal state, overexpression of ZNHIT1 did not alter cellular levels of *α*-Syn, and overexpression of *α*-Syn did not alter cellular levels of ZNHIT1 (Fig. 3D–G). Collectively, these data show that ZNHIT1 regulates basal levels of neurite growth and that overexpression of ZNHIT1 protects against *α*-Syn-induced reductions in cell viability and neurite growth, without affecting cellular levels of *α*-Syn.

### Bioinformatics Analysis Implicates ZNHIT1 Co-expressed Genes in the Regulation of Mitochondrial Function

**Fig. 4:**
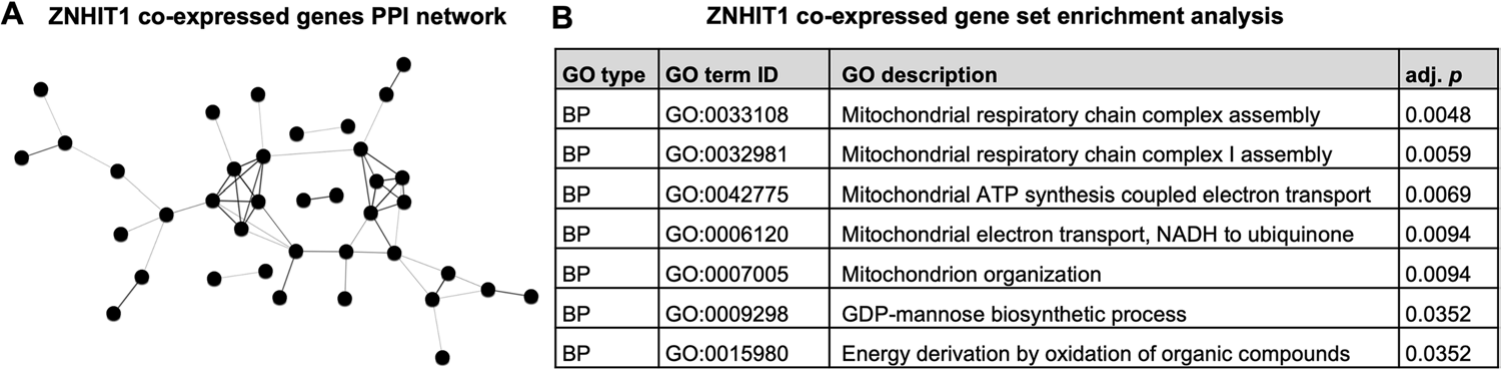
Bioinformatics analysis implicates ZNHIT1 co-expressed genes in the regulation of mitochondrial function.** A** Protein–protein interaction (PPI) network generated from the list of genes that were strongly co-expressed (*r* > 0.7) with *ZNHIT1* in the human SN. **B** Table showing the significantly enriched gene ontology (GO) biological processes (bp) categories resulting from a gene set enrichment analysis of *ZHNIT1* co-expressed genes. Raw data was derived from GSE:60,863. Gene co-expression analysis was performed using the R2 genomics analysis and visualisation platform (https://hgserver1.amc.nl/cgi-bin/r2/main.cgi). The PPI network and GO analysis were performed using STRING (https://string-db.org)

We next sought to gain insight into the cellular processes that may be regulated by ZNHIT1. To do this, we performed pair-wise correlations between *ZNHIT1* and all other genes expressed in the human SN (*n* = 101 samples) using open-source human brain transcriptome data (GSE:60,863) [23]. This analysis identified *n* = 2248 genes that were co-expressed with *ZNHIT1*, after Bonferroni multiple testing correction. We then performed multiple analyses of genes that were strongly co-expressed (*r* > 0.7) with *ZNHIT1* in the SN (*n* = 75) using STRING (https://string-db.org). We first generated a protein–protein interaction (PPI) network to determine if there were any biological relationships among this list of genes strongly co-expressed with ZNHIT1. We used a minimum required interaction score of 0.4 which is a ‘medium confidence’ setting. For the network generated with this list of *ZNHIT1* co-expressed genes, the number of nodes was 75, and the number of edges was 53 (Observed = 53; Expected = 23 from a random list of the same size), with the PPI network having a PPI enrichment *p*-value of 6.28 × 10^−08^ (Fig. 4A). We next performed a gene set enrichment analysis and found a significant enrichment of genes associated with the multiple GO biological processes (bp) categories linked to mitochondrial function (Fig. 4B). These analyses indicate that *ZNHIT1* co-expressed genes are at least partially biologically connected as a group. When considered along with our neurite growth data shown above, this suggests that cellular levels of ZNHIT1 may regulate the cellular response to *α*-Syn-induced impairments in mitochondrial function.

### Bioenergetic State Analysis Shows that Overexpression of ZNHIT1 Prevents α-Synuclein-Induced Impairments in Mitochondrial Function

**Fig. 5:**
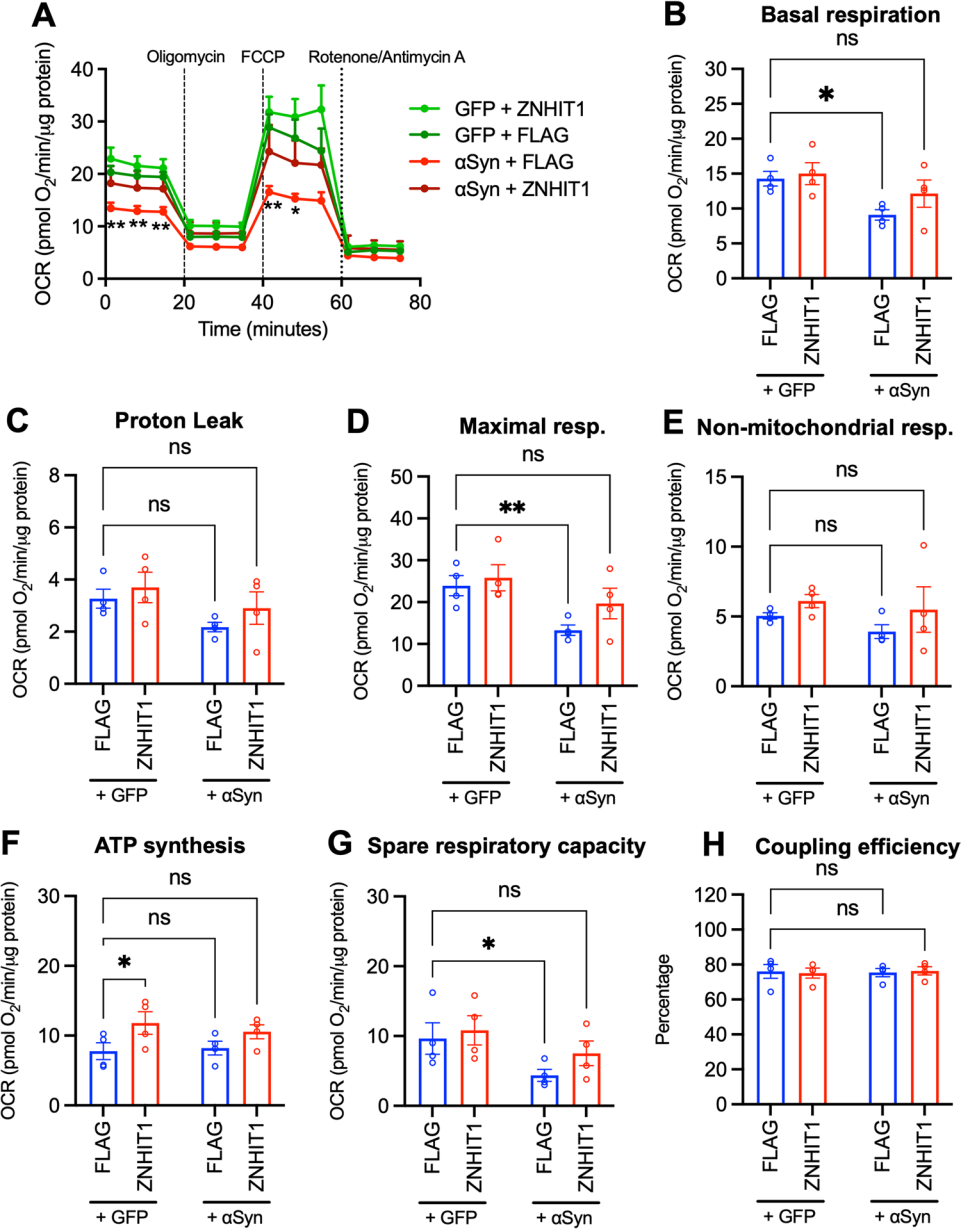
Bioenergetic state analysis shows that overexpression of ZNHIT1 prevents α-synuclein-induced impairments in mitochondrial function. **A** Oxygen consumption rate (OCR) in the Seahorse XF Mito Stress Test in SH-SY5Y cells stably expressing GFP or α-Syn and transfected for 72 h with 500 ng of a plasmid expressing either FLAG (Addgene #31,385) or FLAG-tagged ZNHIT1 (Addgene #15,332). **B**–**G** Graphs of individual parameters of respiration showing **B** basal respiration, **C** proton leak, **D** maximal respiration, **E** non-mitochondrial respiration, **F** ATP production, **G** spare respiratory capacity and **H** coupling efficiency. Data are mean ± SEM of OCR values normalised to protein content per well from *n* = 4 independent replicates. Two-way ANOVA or mixed effects model and post hoc Fisher’s LSD test (*p < 0.05, **p < 0.01 vs FLAG + GFP)

To investigate the hypothesis that ZNHIT1 can modulate the cellular response to αSyn-induced impairments in mitochondrial function, we performed an analysis of cellular bioenergetic state. To do this, we measured the oxygen consumption rate (OCR) and individual parameters of respiration in SH-SY5Y cells stably expressing GFP or α-Syn, and transfected for 72 h with either FLAG as a control or FLAG-tagged ZNHIT1 (Fig. 5A–H). Overexpression of α-Syn without ZNHIT1 overexpression induced a consistent reduction in OCR when compared to the FLAG + GFP group; this was not seen in the other groups (Fig. 5A). We next examined individual parameters of respiration; two-way ANOVA revealed significant main effects of α-Syn on basal respiration (*F*_1,12_ = 8.143, *p* = 0.0145) (Fig. 5B), maximal respiration (*F*_1,12_ = 9.328, *p* = 0.0100) (Fig. 5D), spare respiratory capacity (*F*_1,12_ = 5.512, *p* = 0.0369) (Fig. 5G) and a significant main effect of ZNHIT1 on ATP synthesis (*F*_1,12_ = 6.726, *p* = 0.0235) (Fig. 5F). Post hoc analysis revealed significant impairments in basal respiration (Fig. 5B), maximal respiration (Fig. 5D) and spare respiratory capacity (Fig. 5G) in the FLAG + *α*-Syn group that were not seen in the ZNHIT1+*α*-Syn group. Moreover, higher levels of ATP synthesis were seen in the ZNHIT1 + GFP group compared to the FLAG + GFP control group (Fig. 5F). Collectively, these data show that ZNHIT1 modulates *α*-Syn-impairments in mitochondrial function in SY-SY5Y cells.

## Discussion

In this study, we used gene co-expression analysis to conduct an a priori bioinformatics study to identify genes that are co-expressed with *SNCA* in the human SN [49]. This information formed the basis for subsequent in-depth investigations of underlying molecular events that may be involved in disease pathology in PD. The rationale for this approach is that genes that display significantly correlated co-expression patterns are more likely to be involved in the same cellular pathways and processes, due to their co-regulation [24, 50]. Moreover, co-expression patterns between genes tend to break down in disease states; therefore, such broken correlations can be indicators of pathophysiological molecular dysfunction [24–26]. This type of analysis has been used in many other studies aimed at understanding the molecular basis of cancer [51], schizophrenia [24], chronic fatigue syndrome [52] and Alzheimer’s disease (AD) [25]. Using this approach, we identified a significant correlation between *SNCA* and three genes, *ZNHIT1*, *SAP18* and *HDAC5*, in the SN of control human brain samples. In samples of SN from PD patients, the positive correlation between *SNCA* and *SAP18* was maintained. However, this correlation was lost between *SNCA* and both *HDAC5* and *ZNHIT1*, with the greatest loss observed for *SNCA-ZNHI*T1. As HDAC5 has previously been identified as a regulator of α-Syn-induced impairments in neurite growth [38, 39], this highlights the potential of the approach for identifying genes that may be relevant to α-Syn-induced cellular dysfunction. Although the function of ZNHIT1 in neural cells was largely unknown, it was the top ranked gene in our analysis which indicated a potential link between ZNHIT1 and α-Syn in PD.

ZNHIT1 is a main regulatory component of the SRCAP complex, which is important in the regulation of chromatin remodelling [45, 46]. In our study, we found that overexpression of ZNHIT1 increases deposition of the histone variant H2A.Z in SH-SY5Y cells. This agrees with previous studies showing that ZNHIT1 promotes H2A.Z deposition during myocyte differentiation [40]. Furthermore, other studies have reported that ZNHIT1 levels increase during early differentiation of myocytes and mediate the binding of H2A.Z to chromatin in a p38 MAPK-dependent manner [40]. Although the function of ZNHIT1 in neurons is unknown, H2A.Z deposition has been shown to play a role in heterochromatin regulation [41], DNA repair [42, 43] and transcriptional regulation [44]. Of interest, in LUHMES cells, overexpression of WT or A30P mutant α-Syn leads to downregulation of DNA repair genes [53], which contrasts with its action to stimulate H2A.Z deposition found in our study. We further found that ZNHIT1-induced H2A.Z incorporation is unaffected by overexpression of α-Syn, suggesting that ZNHIT1 may be able to protect against the detrimental cellular effects of α-Syn.

To test this hypothesis, we used α-Syn-induced reductions in SH-SY5Y cell survival and neurite growth as readouts of cellular pathology of relevance to PD. The rationale for this is that neurite degeneration is recognised as a core component of the cellular pathology of PD [1, 54], and further that both overexpression of α-Syn in rodent dopaminergic neurons [17] and mutation of α-Syn in iPSC-derived dopaminergic neurons result in reductions in cell survival and neurite growth [18, 19]. We found that the overexpression of ZNHIT1 significantly increased basal levels of neurite growth and protected SH-SY5Y cells against the adverse effects of overexpression of αSyn-on neurite growth and cell viability. We found that the overexpression of ZNHIT1 did not alter cellular levels of α-Syn and conversely that the overexpression of α-Syn did not affect cellular levels of ZNHIT1 in SH-SY5Y cells. This shows that the beneficial effects of ZNHIT1 overexpression are not secondary to an effect of ZNHIT1 in reducing cellular levels of α-Syn. Collectively, these data identify ZNHIT1 as a novel regulator of neurite growth that protects against the detrimental effects of α-Syn on neurite growth and cell viability.

To gain insight into the gene regulatory networks that may be influenced by ZNHIT1, we used gene co-expression analysis to identify *ZNHIT1* co-expressed genes. This revealed a significant enrichment of genes associated with mitochondrial function in the human SN, suggesting that *ZNHIT1* co-expressed genes are biologically connected as a group and may function as regulators of mitochondrial function. This finding is supported by a previous study showing that ZNHIT1 regulates the expression of genes associated with mitochondrial function during prenatal cardiac development [29]. This is important since mitochondrial dysfunction has been implicated as a key component in the pathogenesis of PD [for review see: 55]. Furthermore, α-Syn has been shown to impair mitochondrial function [see review: 56]. For example, outer mitochondrial membrane proteins, such as voltage-dependent anion-selective channel 1 (VDAC1) [57], translocase of the outer membrane 20 (TOM20) [58] and TOM40 [59], are bound by α-Syn, leading to mitochondrial dysfunction [for review see: 55]. Given the beneficial effects of ZNHIT1 overexpression on neurite growth and cell survival, and the fact that ZNHIT1 co-expressed genes were enriched in those associated with mitochondrial function, this suggested that ZNHIT1 may modulate α-Syn-induced mitochondrial dysfunction.

In agreement with this hypothesis, bioenergetic state analysis revealed that overexpression of α-Syn resulted in a consistent reduction in OCR, as well as significant impairments in basal respiration, maximal respiration and spare respiratory capacity in SH-SY5Y cells stably expressing α-Syn. This agrees with previous studies showing α-Syn-induced impairments in mitochondrial function [for reviews see: 60, 61]. α-Syn has been shown to have a neuroprotective role in maintaining mitochondrial function [60]; however, in disease states such as PD, toxic α-Syn aggregates form as a result of synaptotoxicity and synaptic dysfunction [54], leading to defects in mitochondrial function [for review see: 55]. Interestingly, we found that α-Syn-induced impairments in mitochondrial function were not seen in ZNHIT1-overexpressing cells, suggesting that ZNHIT1 overexpression is sufficient to prevent the effects of α-Syn on mitochondria. Moreover, we found that ZNHIT1 overexpression resulted in a significant increase in ATP synthesis. This finding is consistent with a previous report showing that ATP production was reduced in cardiac tissue of ZNHIT1 knockout mice and that ZNHIT1 was crucial for maintaining the integrity of mitochondrial respiratory complex in cardiac cells [29]. This suggests that ZNHIT1 may be important for maintaining mitochondrial function in dopaminergic neurons in the SN.

In summary, our findings have identified an enrichment of genes co-expressed with *SNCA* in the SN that are involved in histone deacetylation. In particular, we report loss of co-expression of *ZNHIT1* and *SNCA* in PD, which is indicative of functional dysregulation. We also show that ZNHIT1 increases incorporation of the histone variant H2A.Z in SH-SY5Y cells. Functional studies revealed a neuroprotective effect of ZNHIT1 overexpression against α-Syn-induced reductions in neurite growth and cell viability, as well as mitochondrial function, in SH-SY5Y cells. Taken together, our data reveal ZNHIT1 as a potential novel therapeutic target for neuroprotection in PD.

## Supplementary Information

Below is the link to the electronic supplementary material.

Supplementary file1 (PDF 356 KB)

## Data Availability

All data generated during this study are included in this article or are available on reasonable request from the corresponding authors.

## Abbreviations

PD: Parkinson’s disease
α-Syn: α-Synuclein
SNCA: α-Synuclein gene
ZNHIT1: Zinc finger HIT-type containing 1
DA: Dopamine
DAergic: Dopaminergic
NT: Neurotransmission
LB: Lewy body
SN: Substantia nigra
TGFβ: Transforming growth factor β
WT: Wild-type
GFP: Green fluorescent protein
GO: Gene ontology
BSA: Bovine serum albumin
PBS: Phosphate-buffered saline
PBS-T: Phosphate-buffered saline Triton X-100
TH: Tyrosine hydroxylase
ALDH1A1: Aldehyde dehydrogenase 1 family member A1
HDAC5: Histone deacetylase 5
HDAC6: Histone deacetylase 6
SAP18: Histone deacetylase complex subunit SAP18
MORF4L1: Mortality factor 4-like protein 1
H3: Histone 3
ACH3: Acetylated histone 3
AD: Alzheimer’s disease
HAT: Histone acetylase
HDAC: Histone deacetylase
MD: Mitochondrial dysfunction
ROS: Reactive oxygen species
SRCAP: SNF2-related CBP activator protein
VDAC1: Voltage-dependent anion-selective channel 1
MD: Mitochondrial dysfunction
