# Supplementary material for: Gene Co-expression Analysis of the Human Substantia Nigra Identifies ZNHIT1 as an SNCA Co-expressed Gene that Protects Against α-Synuclein-Induced Impairments in Neurite Growth and Mitochondrial Dysfunction in SH-SY5Y Cells
Source: Mol Neurobiol. 2022 Feb 17;59(5):2745–57. doi: 10.1007/s12035-022-02768-9 (PMC9016026; doi:10.1007/s12035-022-02768-9)
Supplement: Supplementary file 1 — Supplementary file1 (PDF 356 KB) [file 12035_2022_2768_MOESM1_ESM.pdf]

Article title: Gene co-expression analysis of the human substantia nigra identifies ZNHIT1 as an *SNCA* co-expressed gene that protects against α-synuclein-induced impairments in neurite growth and mitochondrial dysfunction in SH-SY5Y cells.

Journal name: Molecular Neurobiology

Authors names: Erin McCarthy[1], Aaron Barron[1,2], Noelia Morales-Prieto[1], Martina Mazzocchi[1], Cathal M. McCarthy[2], Louise M. Collins[1,3,5], Aideen M. Sullivan[1,4,5]*, Gerard W. O'Keeffe[1,4,5]*

[1] Department of Anatomy & Neuroscience, University College Cork, Cork, Ireland.
[2] Department of Pharmacology and Therapeutics, University College Cork, Cork, Ireland.
[3] Department of Physiology, University College Cork, Cork, Ireland.
[4] APC Microbiome Ireland, University College Cork, Cork, Ireland.
[5] Parkinson's Disease Research Cluster (PDRC), University College Cork, Cork, Ireland.

***Corresponding authors:**

Gerard W. O'Keeffe g.okeeffe@ucc.ie or
Aideen M. Sullivan a.sullivan@ucc.ie

Supplementary Figure 1

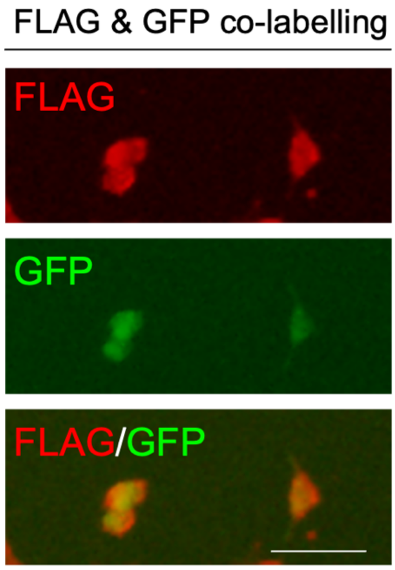

Supplementary Figure 1: SH-SY5Y cells co-express the FLAG tag and GFP when co-transfected. Representative photomicrographs of SH-SY5Y cells transfected with 500 ng of plasmids expressing FLAG-tagged ZNHIT1 together with a plasmid expressing GFP-tagged wild-type α-synuclein (αSyn) and immunocytochemically stained for FLAG (red) to show co-localisation with GFP fluorescence (green) indicating their co-expression in transfected cells.
